# Fabrication and Optimization of a Silodosin In Situ-Forming PLGA Implants for the Treatment of Benign Prostatic Hyperplasia: In Vitro and In Vivo Study

**DOI:** 10.3390/pharmaceutics16111364

**Published:** 2024-10-25

**Authors:** Rabab A. Husseini, Tarek M. Ibrahim, Eslam Hamed, Eman Gomaa, Mennatullah M. Faisal, Ghadeer Wan, Manna Amin, Ali M. Alkolaib, Dina M. Abdelnabi

**Affiliations:** 1 Department of Pharmaceutics, Faculty of Pharmacy, Zagazig University, Zagazig 44519, Egypt; rabab.ahmed@zu.edu.eg (R.A.H.); telmetwally@zu.edu.eg (T.M.I.); emangomaa@zu.edu.eg (E.G.); menna.faisal@zu.edu.eg (M.M.F.); 2 Department of Pharmacology, Faculty of Veterinary Medicine, Zagazig University, Zagazig 44519, Egypt; eslam.hamed@zu.edu.eg; 3 Nanotechnology Research Center (NTRC), The British University in Egypt (BUE), Cairo 11837, Egypt; 4 Department of Pharmaceutical Sciences, Faculty of Pharmacy, Umm Al-Qura University, Makkah 21955, Saudi Arabia; gywan@uqu.edu.sa (G.W.); maamin@uqu.edu.sa (M.A.); 5 Department of Pharmaceutics, Faculty of Pharmacy, Najran University, Najran 11001, Saudi Arabia; amalkolaib@nu.edu.sa

**Keywords:** silodosin, PLGA, in situ-forming implants, I-optimal design, benign prostatic hyperplasia

## Abstract

**Objectives:** Lower urinary tract symptoms (LUTSs) related to benign prostatic hyperplasia (BPH) are common in older men, and alpha-adrenoceptor blockers continue to be a key part of managing these symptoms. This study aimed to formulate injectable poly (lactic-co-glycolic acid) (PLGA) in situ-forming implants (ISFIs) loaded with silodosin (SLD) to address symptoms associated with BPH. This method, which ensures prolonged therapeutic effects of SLD, is intended to decrease dosing frequency and improve treatment outcomes, leading to better patient adherence. **Methods:** An appropriate solvent with favorable PLGA solubility, viscosity, and in vitro release profile was selected. Additionally, an I-optimal design was employed as an optimization technique. An in vivo study in albino male rats was conducted to investigate prostate-specific antigens (PSAs), prostate weight and prostatic index, histopathology, and SLD pharmacokinetics. **Results:** The optimized formulation showed experimental values of 29.25% for the initial burst after 2 h and 58.23% for the cumulative release of SLD after 10 days. Pharmacokinetic data revealed that the SLD–ISFI formulation had lower C_max_ and higher AUC values than subcutaneous (SC) pure SLD and oral commercial SLD capsule, indicating the controlled-release impact and improved bioavailability of the ISFI systems. SLD–ISFI produced a marked drop in the prostatic index by 2.09-fold compared to the positive control. Serum PSA level decreased significantly from 0.345 ± 0.007 to 0.145 ± 0.015 ng/mL after SLD–ISFI injection compared to the positive control. **Conclusions:** This study indicated that the optimized SLD–ISFI formulation proved its efficacy in managing BPH.

## 1. Introduction

Benign prostatic hyperplasia (BPH) refers to the enlargement of the prostate gland caused by the proliferation of prostatic stromal cells. This condition is responsible for various lower urinary tract symptoms (LUTSs) in men, including storage symptoms, such as increased frequency, urgency, and nocturia, and voiding symptoms, such as intermittency and hesitancy. BPH is commonly associated with aging and is prevalent in many men over 40 [1,2,3]. Currently, the treatment options for BPH are pharmacotherapies, including α_1_-adrenoreceptor blockers (α-blockers), β-receptor agonists, anticholinergics, phosphodiesterase-5 inhibitors, 5α-reductase inhibitors, and surgical intervention for severe LUTSs [4,5]. In the lower urinary tract, α_1_-adrenergic receptors are ubiquitous. Human tissues contain three α_1_-receptor subtypes (α_1A_, α_1B_, and α_1D_), with α_1A_ receptors responsible for the smooth muscle tone in the bladder neck and prostate. Therefore, α_1A_ blockers can help improve LUTSs by relaxing the lower urinary tract smooth muscles [6].

Silodosin (SLD) is a selective α_1a_ adrenoceptor blocker, especially for the prostatic α-receptors [7]. It has been proven to be a safe and more effective treatment for urinary tract symptoms in individuals with BPH. It is more effective than tamsulosin and has better cardiovascular tolerance [6,8,9]. The selectivity of silodosin towards α_1a_-receptor blockade is 38 times higher compared to tamsulosin [10]. It has also proven effectiveness as stone-expulsive treatment [3]. SLD is approved by the U.S. Food and Drug Administration and the European Medicines Agency for treating the signs and symptoms of BPH [8,11]. It has a low oral bioavailability of 32% due to extensive first-pass metabolism in the liver [1]. Implantable depot formulations, including poly (lactic-co-glycolic acid) (PLGA) implants, have been designed to bypass hepatic metabolism, which can enhance SLD bioavailability and patient adherence by sustaining SLD release for several days [12,13].

Polymeric implants can either be non-biodegradable or biodegradable based on the polymer used in their fabrication. Non-biodegradable implants must be removed once exhausted, while biodegradable implants have polymers that break down into monomers during and after the release process, which are then metabolized and excreted [14,15,16]. Biodegradable polymers such as poly (lactic acid), poly (glycolic acid), and PLGA are becoming increasingly popular in drug delivery because of their good mechanical strength, easy manufacturing, and wide degradation rates [17]. Moreover, PLGA can offer complete biodegradability and high biocompatibility [18]. The hydrophilic–lipophilic characteristics of PLGA grade are determined by its lactic-acid–glycolic-acid (LA:GA) ratio. As a result, when the percentage of lactide increases, its hydrophilicity and solubility in polar solvents decreases [19].

In situ-forming implants (ISFIs) are becoming more popular as a viable option for controlled-release dosage forms, serving as an alternative to pre-made implants [20]. They offer time- and location-controlled drug release, a straightforward manufacturing process, and a minimally invasive approach [19]. ISFIs can enhance patient adherence [16,21], especially for managing chronic illnesses that necessitate repeated treatments and extended therapeutic periods [14].

ISFI formulations transform into solid depots or gels post-injection in the subcutaneous tissue [16,22]. The transformation mechanisms include chemical [23], physical [24], photo-initiated [25] in situ cross-linking, in situ solidifying organogels [26], or in situ phase separation triggered by temperature change [27], pH change [28], and solvent exchange as in Eligard^®^ ISFIs for the treatment of prostate cancer [29]. Solvent-exchange-based ISFIs, developed by Dunn et al. [30], can be triggered more easily, requiring only water in their environment [22,31,32]. The solvent-exchange-based ISFI system consists of hydrophobic polymers such as PLGA and organic solvents (fully or partially water-miscible) containing the drug. When injected into the body, the solvent diffuses into the adjacent aqueous environment. At the same time, water or tissue fluid, which is not a solvent for the polymer, enters the ISFI, leading to phase separation and the precipitation of the hydrophobic polymer in situ, which can maintain the release of the drug at a steady rate [16,33].

Based on the phase inversion rate, PLGA ISFIs can be categorized as fast- or slow-forming. Highly water-miscible solvents like dimethyl sulfoxide (DMSO) and N-methyl-2-pyrrolidone (NMP) cause rapid-phase inversion, while water-immiscible solvents like triacetin (TA) and ethyl acetate (EA) cause slower-phase inversion. The network formed during matrix solidification is crucial for drug release. The rapid-phase inversion creates large pores close to the surface that lead to high initial burst release from those implants, while slow-phase inversion produces a more dense structure with smaller pores due to limited solvent solubility and slow solvent exchange [22]. The initial burst release of a polymer can also be influenced by its molecular weight and concentration [16].

The current study aims to design SLD-loaded PLGA ISFI formulations using I-optimal response surface methodology to attain an optimized sustained-release formulation of SLD capable of providing suitable SLD blood levels with reduced dosing frequency and enhanced treatment outputs. This, in turn, can enhance patient compliance and adherence to medication. This study also attempted to investigate the effect of the optimized SLD-loaded PLGA ISFI formulation against testosterone-induced BPH in rats.

## 2. Materials and Methods

### 2.1. Materials

SLD was kindly supplied from Al-Esraa Pharmaceutical Optima, Badr, Cairo, Egypt. PURASORB^®^ PDLG 5002A (50% Lactide, molecular weight of 17,000 Da, inherent viscosity of 0.2 dL/g) was purchased from Corbion, Amsterdam, The Netherlands. NMP was purchased from Central Drug House, New Delhi, India. TA was supplied from Euromedex, Souffelweyersheim, France. DMSO and EA were purchased from SD Fine Chem Limited, Mumbai, India. Commercial Cidoteston^®^ ampoules and Flopadex^®^ capsules were purchased from a community pharmacy, Cairo, Egypt. All other chemicals were of analytical grade.

### 2.2. Preliminary Study for the Preparation of ISFIs

#### 2.2.1. Saturation Solubility of SLD in Different Organic Solvents

Saturation solubility of SLD was measured in various organic solvents, including NMP, DMSO, EA, and TA. Excess pure SLD was suspended in each solvent (3 mL) and then shaken in a shaking-water bath at 25 ± 0.5 °C and 100 rpm for 72 h. The samples were then filtered using 0.22 μm syringe filters, and the filtrates were suitably diluted with ethanol and analyzed using a UV–VIS spectrophotometer (Thermo Scientific Spectronic GENESYS 10 UV, Waltham, MA, USA) at λ_max_ 230 nm. Data were presented as mean values (n = 3) ± standard deviation (SD).

#### 2.2.2. Preparation of SLD-Loaded ISFIs

SLD-loaded ISFIs were prepared by dissolving 200 mg of PLGA in various organic solvents to a total weight of 1 g. The mixture was stirred on a hot plate magnetic stirrer at 900 rpm and 60 °C for 30 min in stoppered glass vials. The SLD (40 mg) was then added to the previous mixture with continuous stirring. The prepared formulations were allowed to cool and kept in the refrigerator for further analysis.

#### 2.2.3. Injectability Time Measurement

We used a stopwatch to record each formulation’s injectability time in seconds to measure the force required to push the prepared ISFI through a syringe needle. Each formulation was withdrawn into a syringe with a 21-gauge needle. A 1 kg weight was then placed on the head of the syringe plunger, compressing it in a downward direction, which allowed for the contents of the syringe to be pushed out through the needle [12].

#### 2.2.4. Morphological Study

The structure of ISFIs containing SLD was examined to observe the solidification of the ISFI systems, which relies on the rate of solvent–water exchange after injection into the external aqueous medium [33]. Therefore, the external shapes of ISFIs containing NMP, DMSO, EA, and TA were visually compared and photographs of the formed ISFIs after being injected into phosphate buffer (pH 7.4) at 37 °C after 0.5 and 2 h were captured.

#### 2.2.5. In Vitro Release Study of SLD-Loaded ISFIs

The prepared ISFIs were placed in sealed bottles containing 100 mL of phosphate buffer (pH 7.4). The bottles were placed in a shaking-water bath at 37 ± 0.5 °C and 100 rpm. Samples (3 mL) were taken at predetermined time points on the first day (0.5, 2, 6, 24 h), followed by daily withdrawals for up to 10 days. An equal volume of fresh phosphate buffer (pH 7.4) was added to maintain a constant receptor medium volume. The samples were filtered and analyzed using a UV–VIS spectrophotometer at λ_max_ 230 nm. The results were presented as mean values (n = 3) ± SD.

### 2.3. Experimental Design and Statistical Analysis

Design-Expert^®^ software (version 11, Stat-Ease Inc., Minneapolis, MN, USA) was employed to develop an I-optimal experimental design in the current study. The I-optimal design is better for developing an effective response surface that accurately predicts responses and identifies optimal operating conditions [34]. The I-optimal design is a versatile response-surface design that can accommodate a single numeric and categoric factor, which is impossible with other response-surface designs such as Central Composite and Box–Behnken. It is worth noting that this approach offers a significant advantage in that it is highly adaptable and can be utilized to fit a wide variety of models, including those of the first and second order, as well as quadratic and cubic models [35]. A total of 19 experimental runs were performed comprising two parameters, PLGA amount (A) and type of solvent (B). The studied responses were burst release of SLD after 2 h (Q_2h_) (Y_1_), SLD release after 10 days (Q_10d_) (Y_2_), and injectability time in seconds (Y_3_). Table 1 shows the three levels of the first factor and four categories of the second one. The proposed 19 runs were performed using the abovementioned methods, and the values of Q_2h_, Q_10d_, and injectability time were recorded as previously detailed in the initial investigation. After entering the results of the responses, the following polynomial equation was used to fit the data values:

Y = b_0_ + b_1_A + b_2_B + b_3_AB + b_4_A^2^

where Y is the measured response, (b_0_) is the intercept of the polynomial equation, (b1–b4) are the regression coefficients computed from the observed experimental values of the measured response, (A and B) are the main effects, (AB) is the two-factor interaction (2FI) effect, and (A^2^) is the quadratic interaction effect.

To determine the significance of each coefficient term, an analysis of variance (ANOVA) and *p*-values with a 95% confidence interval (*p* < 0.05) were employed. Various statistical parameters, such as correlation coefficient (R^2^), adjusted R^2^, predicted R^2^, and adequate precision, were compared to ascertain the best-fitting extent of data. Moreover, one-factor plots were generated to analyze the relationships between the studied factors and measured responses.

### 2.4. Selection of an Optimized Formulation and Statistical Validation

The optimization aimed to obtain an optimized SLD-loaded ISFI formulation with minimum burst release Q_2h_, maximum Q_10d_, and minimum injectability time. After conducting the statistical analysis, a numerical optimization method was used to optimize the formulation factors for creating ISFI systems. The desirability value, often employed as a multi-criteria approach, was used to determine the most suitable levels of the factors. This method allowed for achieving a favorable outcome that aligns with the desired criteria for dependent responses shown in Table 1 [36]. A desirability value close to one indicates that the tested response aligns with its ideal value.

### 2.5. In Vitro Characterization of Optimized ISFI Formulation

#### 2.5.1. Injectability Time and In Vitro Release Studies

The injectability time and the in vitro release of the optimized SLD–ISFI were measured as mentioned in Section 2.2.3 and Section 2.2.5 and compared to the predicted values generated by the Design-Expert^®^ software to calculate the percent deviation of actual responses from predicted ones using the following Equation [31]:

$$\mathrm{percent}\;\mathrm{deviation}=\frac{\mathrm{predicted}\;\mathrm{value}-\mathrm{actual}\;\mathrm{value}}{\mathrm{predicted}\;\mathrm{value}}\times 100$$

##### In Vitro Release Kinetics

The in vitro release data of the optimized SLD–ISFI were fitted to different regression equations using DDSolver (China Pharmaceutical University, Nanjing, China) as an Excel add-in that provides a wide range of kinetic release models (zero order, first order, Higuchi, and Hixson–Crowell), as well as a Korsmeyer–Peppas model, and generates parameters such as the release rate constant (K) and release exponent (n), along with the R^2^ values [37]. The higher the R^2^, the greater the goodness of fit for the model. This can reduce the time and error in selecting an appropriate release model [38]. The equations of those models are as follows:

Zero-order model: Q_t_ = K_o_·t,

First-order model: Q_t_ = 1 − e^−Kt^,

Higuchi model: Q_t_ = K_H_.t^1/2^,

Hixson–Crowell: Q_o_^1/3^ − Q_t_^1/3^ = K_HC_.t, and

Korsmeyer–Peppas model: Q_t_/Q_∞_ = K_KP_.t^n^

where Q_t_ is the amount of drug released at time (t), Q_o_ is the initial amount of drug released, and Q_∞_ is the amount of drug released at time infinity (∞). K_o_, K, K_H_, K_HC_, and K_KP_ are the release rate constants of the previous models, respectively, and n is the release exponent. The “power law” model of Korsmeyer–Peppas describes the drug release mechanism by n value.

#### 2.5.2. Scanning Electron Microscopy (SEM)

The optimized SLD–ISFI formulation’s internal structure and external surface were examined using a scanning electron microscope. The formulation was injected into stoppered glass bottles containing a phosphate buffer (pH 7.4) and then placed in a shaking-water bath at 37 ± 0.5 °C and 100 rpm. Samples of the formed implants were collected at different time points (2 h, 1, and 10 days) and allowed to air-dry at room temperature. Afterwards, the samples were thinly spread on aluminum stubs and gold-coated using a sputter coater under a high vacuum. Finally, SEM photomicrographs were taken at a magnification power of 250× and an accelerating voltage of 30 kV.

#### 2.5.3. Fourier Transform Infrared (FTIR) Spectroscopy

The FTIR spectra of pure SLD powder, PLGA, optimized SLD-free ISFI, and the optimized SLD–ISFI formulation were scanned with an FTIR (FTIR spectrophotometer, PerkinElmer, Inc., Waltham, MA, USA). The analysis covered the range of 4000–500 cm^−1^.

#### 2.5.4. Differential Scanning Calorimetry (DSC)

The thermal behaviors of the pure SLD powder, PLGA, optimized SLD-free ISFI, and optimized SLD–ISFI formulation were studied using DSC (DSC-60, Shimadzu, Kyoto, Japan). The samples were placed in sealed aluminum pans and subjected to a heating range of 25–200 °C at a consistent heating rate of 10 °C/min and a nitrogen flow rate of 30 mL/min.

### 2.6. In Vivo Evaluation of the Optimized SLD–ISFI Formulation

#### 2.6.1. Animals and Ethical Approval

Male albino rats weighing 200~250 g were utilized for the study. They were procured from an animal-breeding center at Zagazig University in Egypt. Before commencing the experiments, the rats were allowed to adapt for a week. They were housed in a room with a controlled temperature, subjected to a 12 h light and 12 h dark cycle, and given unrestricted access to food and water. The experiments were conducted according to the Institutional Animal Care and Use Committee (IACUC) guidelines of the Faculty of Pharmacy, Zagazig University (Approval number: ZU-IACUC/3/F/390/2023).

#### 2.6.2. Induction of BPH and Animal Groups

For induction of BPH in rats, they received 3 mg/kg testosterone oenanthate (Cidoteston^®^) subcutaneously (SC) five days a week for two weeks [39].

Animals were randomly assigned to 5 experimental animal groups (n = 6) as follows:

Group I: control group, which was not administered any treatment or testosterone.

Group II: positive control group (BPH-induced), which was not administered any treatment.

Group III: received a SC pure SLD suspension at a daily dose of 0.82 mg/kg [40] within 1 h before testosterone administration for 10 days.

Group IV: received a daily dose of 0.82 mg/kg of suspended commercial oral SLD capsules (Flopadex^®^) within 1 h before testosterone administration for 10 days.

Group V: received a single SC injection of the optimized SLD–ISFI containing 8.2 mg/kg within 1 h before testosterone administration.

#### 2.6.3. High-Performance Liquid Chromatography (HPLC) Analysis

An HPLC instrument (San Jose, CA, USA) equipped with a C18 column (Hypersil gold, San Jose, CA, USA) of a 4.6 mm × 250 mm, 5 μm particle size column was used for the drug analysis in rats’ plasma samples. The method was conducted according to Kishore, 2012 [41] with slight modifications. The mobile phase consisted of acetonitrile and buffer (1 mL triethylamine in 1000 mL water) (50:50 *v*/*v*). The final pH was adjusted to 3 with ortho-phosphoric acid. The injection volume was 20 µL, and the flow rate was 1 mL/min. The HPLC assay was performed at 225 nm using a photodiode array detector. The mobile phase was degassed by sonication.

#### 2.6.4. Pharmacokinetic Study

To determine the blood levels of SLD, blood samples were collected in heparinized tubes at 0.5, 1, 2, 3, 4, 6, and 24 h for groups II, III, IV, and V then at 2, 3, 4, 5, 6, 7, and 10 days for only groups II and V. The collected blood samples were centrifuged for 10 min at 3000 rpm. The SLD concentration in each plasma sample was calculated after constructing the calibration curve of SLD in the plasma using HPLC. The pharmacokinetic parameters of SLD from the in vivo study were calculated using PK solver Excel add-in based on the non-compartmental analysis method [42]. These parameters included the maximum plasma concentration (C_max_), the time needed to reach the C_max_ (T_max_), the apparent elimination rate constant (K_el_), the areas under the curve (AUC_0-t_ and AUC_0–∞_), and the area under the first-moment curve (AUMC_0–∞_). The elimination half-life (t_1/2_) and the mean residence time (MRT) were also calculated. The pharmacokinetic parameters were evaluated using SigmaPlot version 14.5 through one-way ANOVA statistical analysis.

#### 2.6.5. Determination of Prostate-Specific Antigen (PSA)

Serum samples from rats were collected on the final day of the experiment and used to measure the serum levels of PSA using ELISA Kits that were designed for rats’ PSA according to the manufacturer’s instructions (Hoffmann-La Roche Ltd., Basil, Switzerland) [43]. To assess the significant difference between the SLD–ISFI and the positive control group, a one-way ANOVA test using SigmaPlot version 14.5 was employed.

#### 2.6.6. Prostatic Index

The rats were euthanized 72 h after the final testosterone dose, and their prostatic tissue was dissected and weighed. The prostatic index for each rat was computed by dividing the prostatic tissue weight by the body weight (g/g) [39].

#### 2.6.7. Histopathology and Histomorphometric Analysis

The prostatic tissue samples were cut, exposed to ethanol, and assembled into paraffin blocks. Histological sections (10–15 μm) were cut with a cryostat (Leica) and subjected to routine staining techniques using hematoxylin and eosin (H and E). After staining, a minimum of three sections were photographed under the microscope [44]. These images were further analyzed for histomorphometric analysis using image analysis software (ImageJ, 1.54 g, NIH, Bethesda, MD, USA) to examine the height and the number of projections per acini [39].

## 3. Results and Discussion

### 3.1. Preliminary Studies of SLD-Loaded ISFIs

SLD’s solubility was tested in NMP, DMSO, EA, and TA. Those solvents were selected because they are considered safe additives in parenteral products for human use due to their high median lethal doses [12]. NMP and DMSO are commonly used in injectable human products, while TA is used in oral dosage forms. EA is classified as an International Conference on Harmonization (ICH) Class 3 solvent, meaning it is less toxic and poses lower risks to human health [45]. In this study, the SLD solubility in NMP, DMSO, EA, and TA solvents was found to be 674.63 ± 1.12, 580.14 ± 1.39, 73.40 ± 2.86, and 79.21 ± 1.09 mg/mL, respectively (Table 2). These results showed that SLD has higher solubility in water-miscible solvents (NMP and DMSO) than in water-immiscible solvents (EA and TA). Moreover, the PLGA (50:50) solubility was estimated to be good in all solvents; however, the dissolution was slower in TA compared to other solvents. The high molecular volume (obtained by dividing the molecular weight by the solvent’s density) and its high viscosity compared to other solvents may be the reason for this observation [46]. This finding aligns with Ibrahim et al. [31] and Camargo et al. [46], who demonstrated that the solvents with smaller molecular volumes, such as NMP (96.5 mL/mol), DMSO (71.03 mL/mol), and EA (97.68 mL/mol), can dissolve polymers more quickly than those with larger molecular volumes, like TA (188.2 mL/mol), which dissolves them to a lesser extent and at a slower rate. Another critical factor influencing PLGA solubility in the solvent is the solvent’s Hildebrand solubility parameter (δ). The optimal solubility of PLGA was observed by Lambert and Peck, who found that it was the highest in solvents with solubility parameters ranging from 18.41 to 22.50 MPa^1/2^ [47,48]. Table 2 demonstrates that the solubility parameters of all solvents were either within or near this range, resulting in good PLGA dissolution, especially the used PLGA (50:50), which has a low molecular weight, intrinsic viscosity (0.2 dL/g), and was added at only a 20% concentration [48].

**Figure 1:**
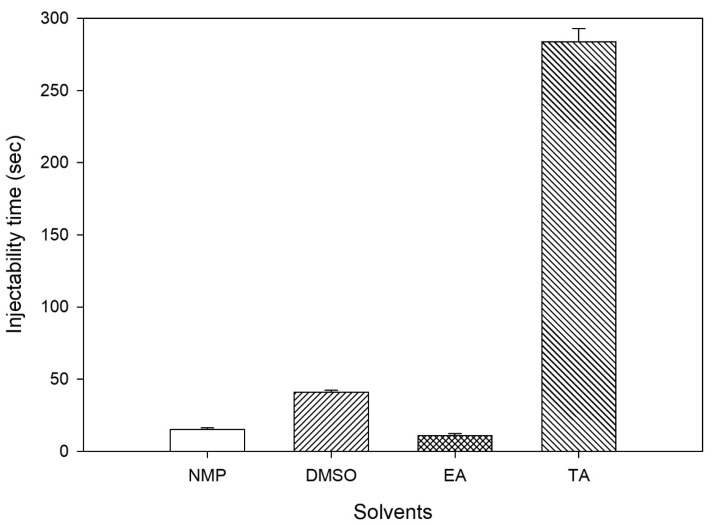
Time required for injecting 1 g of ISFIs containing 200 mg of PLGA from a syringe needle using different solvents.

The ability of polymer solutions to be injected is essential for formulating ISFIs. This means the polymer solution utilized in creating ISFIs should flow smoothly through the needle/syringe without causing a blockage or meeting high resistance [51]. The time required to inject the developed formulations through a syringe needle is demonstrated in Figure 1. Due to the constant concentration of PLGA (20%) in the four preliminary formulations, differences in injectability were attributed to variations in solvent viscosity; in fact, the injectability time was reduced as the solvent’s viscosity decreased. Low viscosity solvents, NMP, DMSO, and EA, had injectability times of 15.17 ± 1.00, 41.05 ± 1.22, and 10.84 ± 1.45 s, respectively. However, TA formulation required increased time for injection through the syringe needle (283.75 ± 8.98 s). The higher viscosity of TA compared to other solvents could be the reason for this, as shown in Table 2.

**Figure 2:**
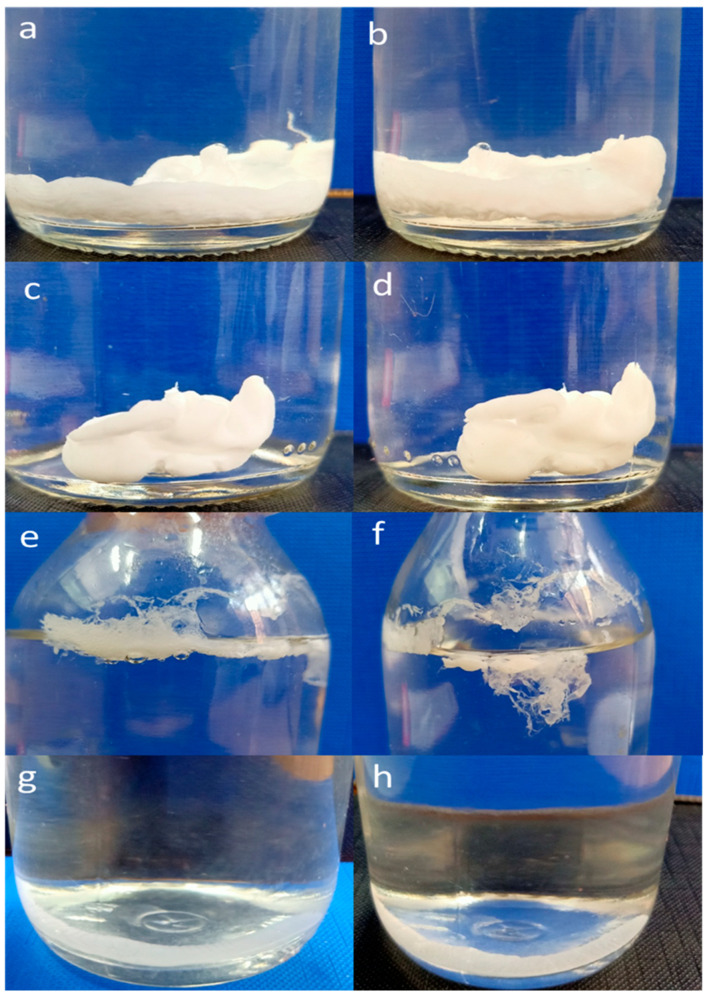
Morphological observation of the ISFIs after 0.5 and 2 h injection of NMP (**a**,**b**), DMSO (**c**,**d**), EA (**e**,**f**), and TA (**g**,**h**) polymeric solutions into phosphate buffer (pH 7.4).

In order to create an ISFI formulation, it is essential that the polymer solution dissolves well and is easily injectable. Additionally, the solution should be able to form a semi-solid implant upon contact with water, which causes phase separation and solidifies the implant [33,51]. As demonstrated in Figure 2, different external shapes of the ISFIs were visually observed upon injection into the buffer, with variations depending on the type of solvent used. Rapid formation of a smooth-surfaced, compact depot within 0.5 h using hydrophilic solvents (NMP and DMSO) was noted. This could be attributed to the quick extraction of the water-miscible solvent into the external medium, leading to a swift transition from solution to gel. Subsequently, the hydrophobic polymer precipitated rapidly, forming the matrix depot [52]. Moreover, after 2 h, there was no notable variation in the structure of the formed depot, which indicated the complete formation of the depot once the polymer solution encountered the buffer.

In contrast to the depot formation observed in the water-miscible systems, the hydrophobic solvent (EA) resulted in the formation of a film at the water surface within 0.5 h, as illustrated in Figure 2. Upon injection, the polymer/EA solution formed tiny droplets that quickly rose and coalesced to create a distinct layer on the water surface, ultimately resulting in a film with an irregular appearance instead of a depot. This phenomenon could be attributed to the hydrophobic nature of EA, an organic solvent that is immiscible with water and has a lower density [51]. Moreover, injecting the polymer/TA solution into the buffer did not result in an immediate solution-to-gel transition. This is potentially due to the solvent’s lower water miscibility, resulting in slower diffusion into the aqueous phase. After 2 h, a more dense depot was formed due to the precipitation of the polymeric solution due to the diffusion of more solvent over time [52].

**Figure 3:**
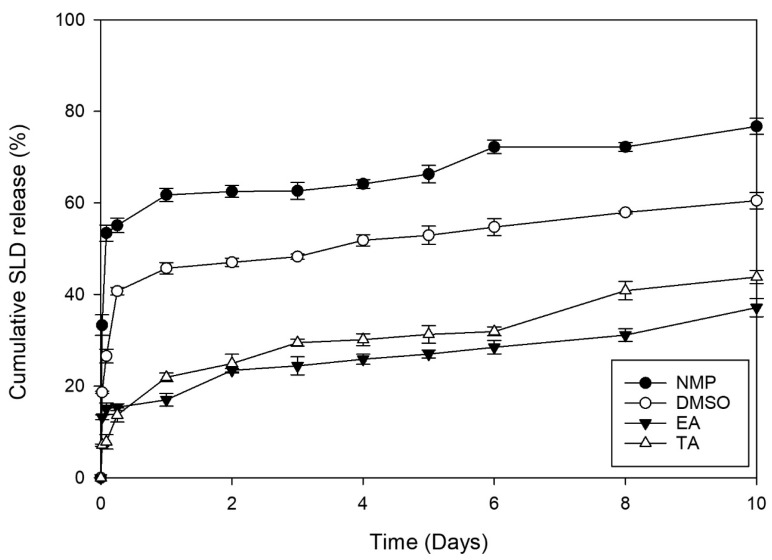
Preliminary in vitro release of SLD (mean ± SD, n = 3) from ISFIs containing 200 mg PLGA in NMP, DMSO, EA, and TA.

In vitro drug release from PLGA ISFIs can be monophasic, bi-phasic, or tri-phasic. The tri-phasic release usually comprises an initial burst release, a plateau diffusion phase, and a fast release phase due to PLGA erosion [19]. Figure 3 illustrates the release of SLD from ISFIs containing 200 mg PLGA in NMP, DMSO, EA, and TA for 10 days. The four ISFIs demonstrated a bi-phasic release profile with an initial burst and a slower, diffusion-controlled release phase.

The solvent’s characteristics significantly affected the drug’s initial release and diffusion after the ISFIs solidified in the external medium [33]. The release of SLD during the first 24 h from EA and TA was significantly lower than that observed in formulations containing NMP or DMSO, as demonstrated in Figure 3. The rapid diffusion of hydrophilic organic solvents, such as NMP and DMSO, towards the external aqueous buffer could explain the higher SLD burst release observed, leading to a rapid solution-to-gel conversion and the formation of a polymer network with increased access of the drug to the implant surface [19,52,53]. In contrast, the initial burst release of SLD from EA and TA ISFIs on the first day was markedly lower than those containing other organic solvents. Systems containing hydrophobic solvents, like EA and TA, usually show slow solution-to-gel conversion, forming an ISFI structure with low porosity. Such a structure can reduce the diffusivity of drugs, leading to a decrease in the initial burst release of the drug [19,53]. Hence, creating desirable burst drug release profiles from ISFIs can be achieved by combining water-miscible (hydrophilic) and -immiscible (hydrophobic) solvents. This approach also helps maintain appropriate viscosity and phase inversion. Owing to the undesirable irregular appearance of EA-containing ISFI (Figure 2), EA solvent was excluded, while NMP, DMSO, and TA were selected for further investigations. In addition, a 1:1 mixture of NMP (showing the highest burst release) and TA (showing the lowest burst release) was also studied in the further design studies.

### 3.2. I-Optimal Experimental Design

#### 3.2.1. Experimental Setup and Model Selection

**Figure 4:**
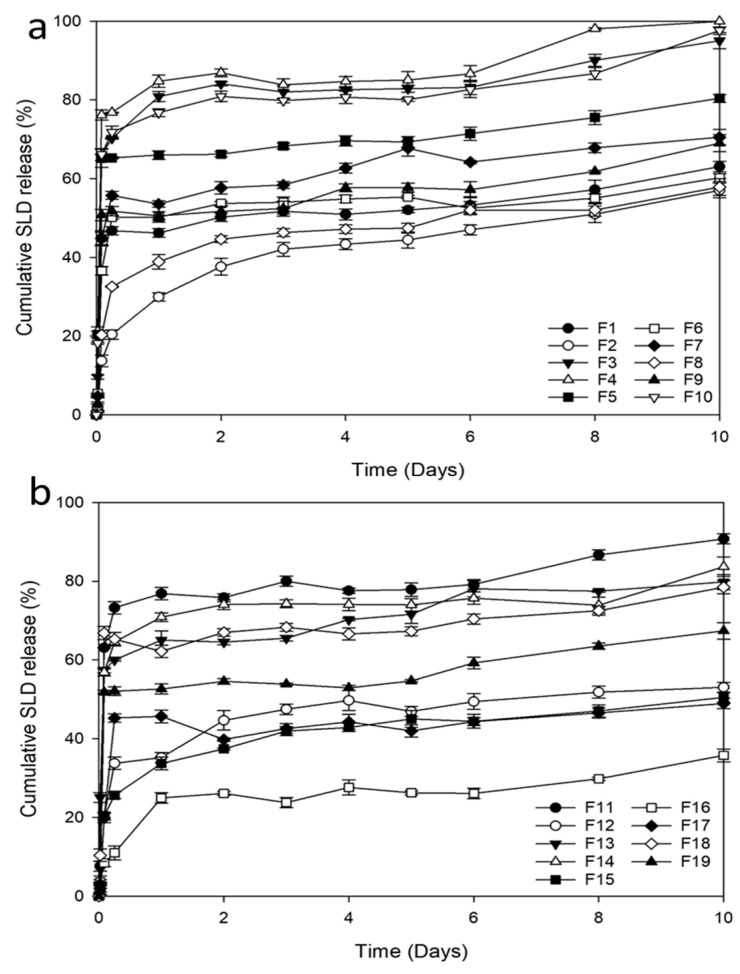
In vitro release profiles of SLD from ISFI formulations: (**a**) F1–F10 and (**b**) F11–F19.

A total of nineteen runs generated by the I-optimal design were prepared, and their responses were evaluated according to changing the independent factors. The composition and recorded results for each of the nineteen formulations can be found in Table 3. The SLD in vitro release profiles from these formulations are shown in Figure 4. The chosen ranges for PLGA amount and the solvents studied led to a wide range of response results. The Q_2h_ ranged from 8.46% to 76.15%, Q_10d_ ranged from 26.62% to 88.56%, and the injectability time ranged from 3.42 s to 210.75 s. The formulations’ observed responses were examined using Design-Expert^®^ software, and polynomial equations were obtained from the data to describe the main effects and interaction effects.

Predictive statistical models were developed based on the factors that significantly impacted each response, as suggested by ANOVA results (*p* < 0.05) (Table 4). These models were created by fitting combinations of these variables in polynomic models up to the third order, using stepwise backward regression to optimize their values. The three models for (Y1–Y3) were found to be significant due to their low *p*-values (<0.0001) and high F-values that indicate they did not occur due to noise (43.36, 88.84, and 758.07, respectively). All models displayed an insignificant lack-of-fit (*p* > 0.05) relative to the pure error and had satisfactory SD values. The models also had acceptable R^2^, and the differences between predicted and adjusted R^2^ values were within acceptable limits (R^2^ pred − R^2^ adj < 0.2) (Table 4) [54]. Moreover, the models’ polynomial equations could determine each factor’s effect on the responses based on the sign of the coefficient. A positive sign could indicate a synergistic effect, while a negative sign could suggest an antagonistic effect [55]. The equation for each model was as follows:

Y_1_ = 43.30 − 16.02 A + 21.14 B[1] + 0.54 B[2] − 23.53 B[3] + 9.22 AB[1] − 3.99 AB[2] + 1.98 AB[3]

Y_2_ = 59.34 − 10.99 A + 19.91 B[1] − 0.13 B[2] − 17.81 B[3]

Y_3_ = 42.83 + 37.50 A − 35.25 B[1] − 26.39 B[2] + 66.34 B[3] − 30.27 AB[1] − 22.51 AB[2] + 58.40 AB[3] + 4.29 A^2^

After fitting the data for Y_1_, the 2-factor interaction (2FI) model was found to be the best fit. According to the polynomial equation, factors A (PLGA amount) and B (solvent type) significantly influenced the burst release. There were significant interaction effects between factors (A and B); however, the quadratic term (A^2^) showed no significant effect on the burst release (Table 4). The equation of the Y_2_ model displayed a significant effect of A and B on the Q_10d_. Because the Y_2_ model was linear, no interaction or quadratic effects were observed (Table 4). The polynomial equation for Y_3_ showed a significant effect of A, B, the interaction term (AB), and the quadratic term (A^2^) on the injectability time (Table 4).

#### 3.2.2. Effects of Independent Factors on the Q_2h_ (Y_1_)

**Figure 5:**
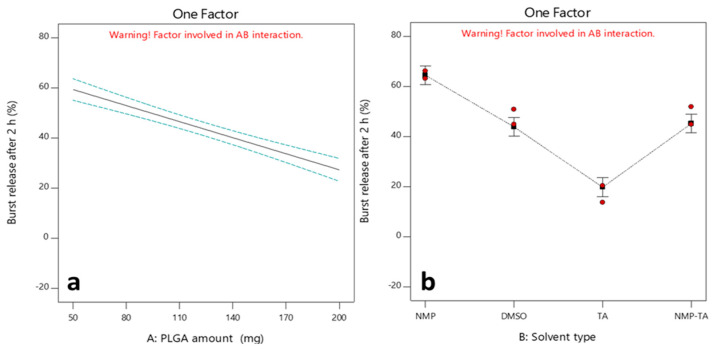
One-factor plots showing the effects of factors A: PLGA amount (**a**) and B: Solvent type (**b**) on Y_1_.

Figure 5a shows that the burst release of SLD decreased by increasing the PLGA amount from 50 to 200 mg. Formulations with a greater quantity of PLGA exhibited substantially reduced initial burst releases compared to those with a lesser amount of PLGA polymer. When the polymer concentration is higher, more polymer is available to fill the interior of the ISFIs, resulting in smaller cavities and increasing the thickness of the polymer shells. This resulted in the drug having to travel a greater distance through the PLGA matrices and slowed down the solvent/water exchange [56]. In addition, increased hydrophobicity of the matrix due to increasing the PLGA amount also contributed to retardation of the water uptake, resulting in a decrease in the initial drug release into the external aqueous buffer [57].

Figure 5b shows that the initial burst release of SLD from TA-containing ISFIs (20.36%) was significantly lower than that of those containing NMP (66.11%) or DMSO (44.82%) at the medium PLGA concentration (125 mg). As proven previously in the preliminary in vitro release, DMSO and NMP, which are hydrophilic organic solvents, showed a higher initial release, likely due to their quick diffusion into the aqueous buffer, leading to a rapid phase inversion system and higher initial release of SLD. However, systems containing hydrophobic solvents, like TA, usually are slow-phase inverting systems, forming an ISFI structure with low porosity that can reduce the SLD diffusion, decreasing the initial burst release [19,52]. Even though NMP and DMSO solvents are both miscible in water and typically undergo rapid phase inversion, they demonstrated varying potential for the initial burst release of SLD after 2 h. NMP forms a depot quickly due to its high miscibility with water, but it is highly porous and permeable compared to DMSO [58]. In addition, DMSO was reported to require less water for precipitation, leading to a more rapid solidification rate, thus efficiently locking the drug dissolved in the system and resulting in a lower initial release [59]. It is worth noting that increased SLD solubility in the NMP compared to DMSO can lead to a higher burst release due to elevated free drug within the system [12,59]. As predicted, mixing NMP and TA resulted in a burst release between both and close to DMSO. The NMP solvent, which is hydrophilic, exits the polymer solution close to the surface rapidly, resulting in the rapid formation of the depot. Still, the quick influx of water into the matrix is limited by the hydrophobic solvent (TA), which slows the release of solvent, prolongs the phase inversion, and ultimately reduces the burst release [60,61].

#### 3.2.3. Effects of Independent Factors on the Q_10d_ (Y_2_)

**Figure 6:**
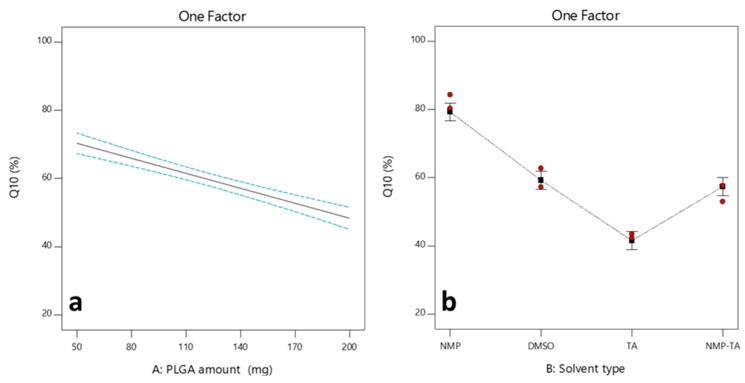
One-factor plots showing the effects of factors A: PLGA amount (**a**) and B: Solvent type (**b**) on Y_2_.

As demonstrated in Figure 6a, increasing the amount of PLGA reduced the Y_2_ response. The formulations containing a greater amount of PLGA exhibited a substantially reduced overall release of SLD after 10 days compared to those containing a smaller quantity of PLGA polymer. This may be due to the increase in PLGA molecules relative to drug molecules, which could improve PLGA’s capacity to bind a greater number of SLD molecules. Consequently, a smaller proportion of the drug would escape into the external aqueous buffer, resulting in a more sustained release of the drug [62]. Moreover, the increased PLGA/drug ratio in ISFI systems resulted in increased polymer–polymer interaction, less porous matrix, and higher viscosity. As a result, the water uptake and rate of drug diffusion were reduced, resulting in sustained and cumulative drug release [19,31]. This was supported by results that showed a decrease in the burst release of the drug with the increased amount of PLGA. Moreover, the effect of the solvent on the cumulative SLD released after 10 days is shown in Figure 6b. At medium PLGA amount (125 mg), the cumulative SLD released were 84.26%, 62.69%, 42.22%, and 57.55% using NMP, DMSO, TA, and NMP–TA, respectively. The cumulative release from ISFIs prepared with NMP and DMSO was higher due to the fast matrix formation, resulting in a sponge-like implant with a porous structure that helped diffuse the drug and water. The TA-containing ISFI formation was slower, leading to more dense, less porous implants and slower SLD release [63]. The addition of a low water-miscible solvent, such as TA, to NMP systems results in a reduction in the cumulative SLD release by approximately 27%.

#### 3.2.4. Effects of Independent Factors on the Injectability Time (Y_3_)

**Figure 7:**
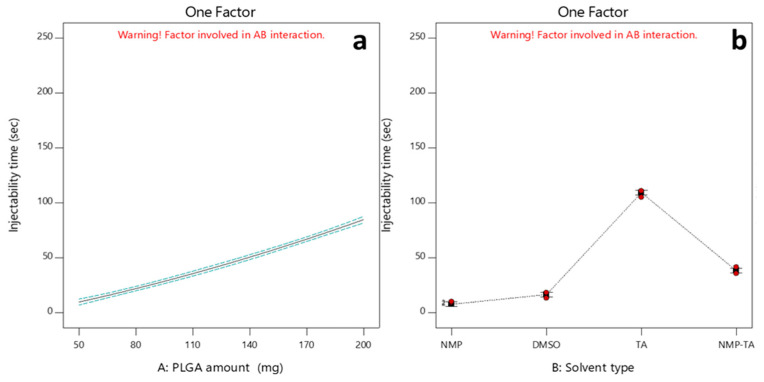
One-factor plots showing the effects of factors A: PLGA amount (**a**) and B: Solvent type (**b**) on Y_3_.

The duration required to push the ISFI’s developed formulations through the syringe needle after preparation ranged between 3.42 and 210.75 s, as indicated in Table 3. The time it takes for the ISFI formulations to be injected was found to increase as the PLGA concentration increased. (Figure 7a). This observed phenomenon could be due to the fact that the viscosity of the ISFI formulations is directly related to the concentration of PLGA [60]. Moreover, it was observed that all tested solvents had an adequate solvating potential for the PLGA during the ISFI’s development (Table 2). However, injection of TA formulations required more time and force (Figure 7b). This could be attributed to the higher viscosity of TA (17.4 cP) compared to other solvents (2 cP for DMSO and 1.65 cP for NMP) (Table 2).

#### 3.2.5. Optimization Process and Statistical Validation

**Figure 8:**
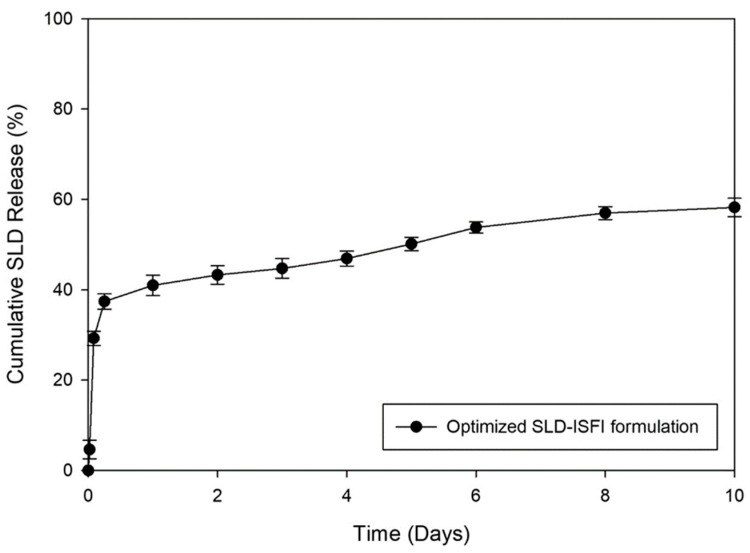
In vitro release of the optimized SLD-loaded ISFI formulation (mean ± SD, n = 3).

The variables studied were adjusted to meet specific goals for the measured responses, as described in Table 1. The necessary criteria involved were reducing the initial burst release of SLD from the ISFIs after 2 h (Q_2h_), increasing its cumulative release over 10 days (Q_10d_), and minimizing the time required for injectability. This optimization resulted in an optimized formulation containing 156.4 mg of PLGA using DMSO as a solvent with the highest desirability value of 0.626. According to the software, the predicted values for Y_1_, Y_2_, and Y_3_ responses were 35.46%, 54.60%, and 23.46 s, respectively. After preparing the optimized formulation, the in vitro release study and the injectability test were conducted. The study results showed that the experimental values for Y1, Y2, and Y3 responses were 29.25%, 58.23% (Figure 8), and 21.59 s, respectively. The percentage deviation values were below 5%, indicating that the estimated and actual values were closely aligned. This suggests that the optimization tools used in the study were effective in predicting and explaining the impact of the factors studied on the measured responses. As a result, the models were considered to be valid, dependable, and suitable for accurately depicting the effects of the studied factors on the responses.

Different mathematical models were examined and compared in Table 5 to determine how SLD was released from the optimized ISFI formulation. The best model for describing the mechanism of drug release was found based on the highest R^2^ value. The release profile of the optimized SLD–ISFI formulation was best described by the Higuchi model equation (R^2^ = 0.9113), indicating that SLD release is diffusion-controlled [64]. In addition, the Korsmeyer–Peppas model showed an n value of 0.233 (less than 0.5). This indicates that the release of the drug is consistent with the Fickian diffusion mechanism (case I transport), in which the drug diffuses from high- to low-concentration areas [65].

### 3.3. In Vitro Characterization of the Optimized ISFI Formulation

#### 3.3.1. SEM Study

**Figure 9:**
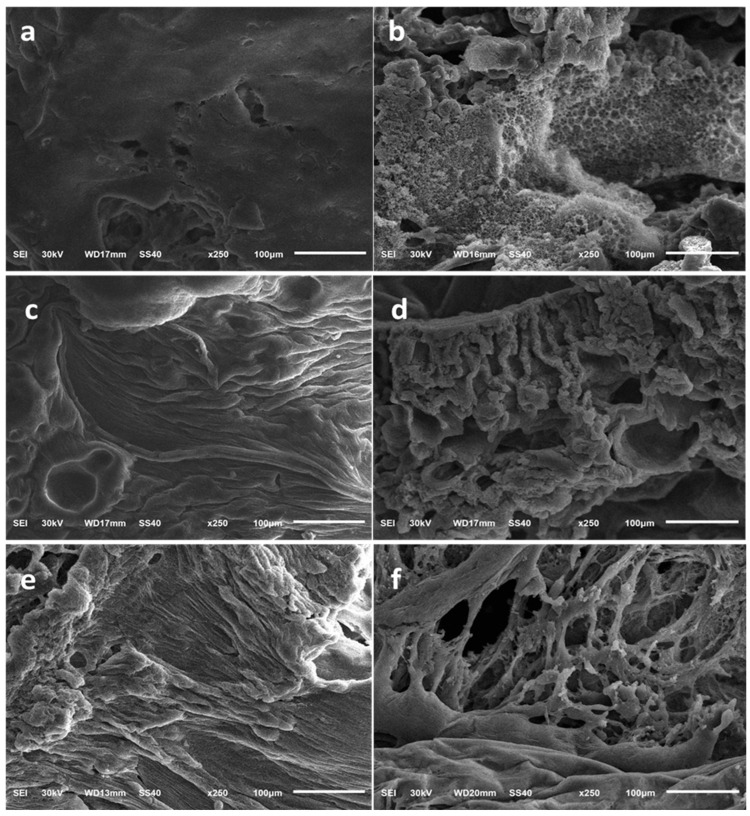
SEM study of the optimized ISFI formulation: (**a**) external surface after 2 h, (**b**) internal structure after 2 h, (**c**) external surface after 1 day, (**d**) internal structure after 1 day, (**e**) external surface after 10 days, and (**f**) internal structure after 10 days.

The optimized ISFI formulation’s internal and external morphology was observed using a scanning electron microscope. The formulation was retrieved from the buffer after 2 h, 1-, and 10 days to inspect its morphology. After 2 h, the optimized ISFI external surface (Figure 9a) and internal structure (Figure 9b) showed a uniform and smooth appearance without pores. The surface of the formulation displayed some cracks after 1 day, with an increase in the thickness of the external shell (Figure 9c). This could be due to the DMSO’s high affinity for water, resulting in fast phase inversion and more polymer precipitation at the surface during the solvent exchange phase [66]. Additionally, the internal structure of the ISFI appeared slightly spongy, with some visible gaps (Figure 9d), which could result from the fast solvent exchange [67]. Over time, the gaps grew bigger, while the thickness of the polymer shell decreased. The surface became highly wrinkled and irregular after 10 days (Figure 9e). The cross-section revealed a rising quantity of internal voids that were growing in size (Figure 9f), indicating the beginning of bulk erosion of the PLGA polymer and the increased release of SLD from the optimized ISFI [31,67].

#### 3.3.2. FTIR Study

**Figure 10:**
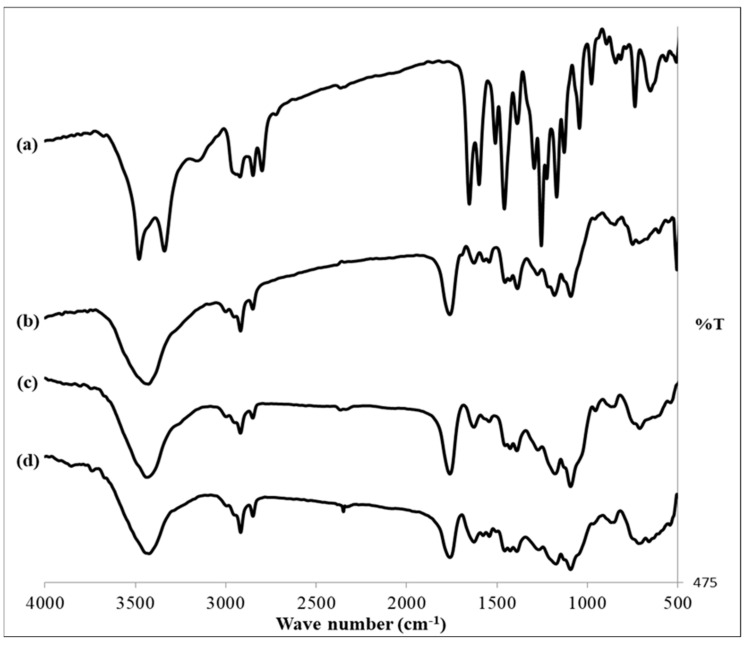
FTIR spectrums of (a) pure SLD, (b) PLGA, (c) blank ISFI formulation, and (d) optimized ISFI formulation.

The chemical interactions between the drug and the components in formulating the ISFI system were studied using FTIR analysis. This was conducted by detecting any shift or absence of spectral peaks, as depicted in Figure 10. The characteristic peaks in the FTIR spectrum of SLD are 3384 cm^−1^, 3201.61 cm^−1^, 2941.24 cm^−1^, 1630 cm^−1^, and 1508 cm^−1^, corresponding to O–H stretching, N–H stretching, C–H bending, C=O stretching, and C=C bending, respectively [68]. The PLGA spectrum displayed peaks at 3483 cm^−1^ (O–H stretching), 2998, 2852 cm^−1^ (C–H stretching), 1751 cm^−1^ (C=O stretching), 1457 cm^−1^ (C–H bending), and 1092 cm^−1^ (C–O stretching) [69,70]. The spectrum of the blank ISFI formulation showed a broad peak at 3480 cm^−1^ and a sharp peak at 1757 cm^−1^, which represented characteristic peaks of PLGA. The distinctive peaks of PLGA were observed in the FTIR spectrum of the optimized ISFI formulation, without any new peaks, suggesting that there are no chemical interactions between the drug and excipients [71].

#### 3.3.3. DSC Study

**Figure 11:**
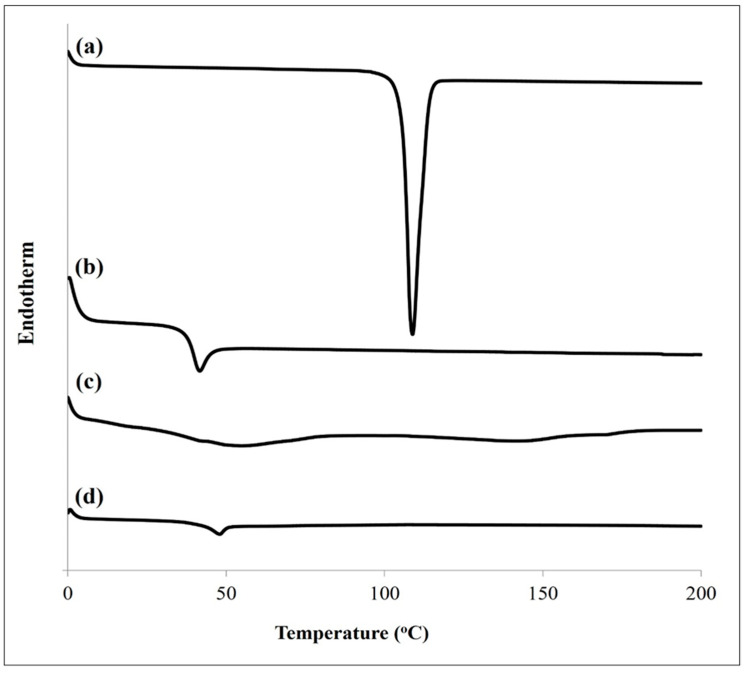
DSC thermograms of (a) pure SLD, (b) PLGA, (c) blank ISFI formulation, and (d) optimized ISFI formulation.

As demonstrated in Figure 11, pure SLD showed a sharp endothermic peak at 108.9 °C, corresponding to the melting point of its crystalline form [1]. The PLGA showed a glass transition temperature of 41.18 °C with no melting peak. An endothermic event accompanied the glass transition temperature of the PLGA due to polymer relaxation [72]. The ISFI formulation, which lacked the SLD, displayed a glass transition temperature that matched that of the PLGA polymer. There were no discernible differences in the thermal properties between the optimized ISFI formulation and the blank formulation, as the SLD’s distinctive melting peak was absent. This affirmed that the SLD was miscible with the PLGA and had transitioned into an amorphous state.

### 3.4. In Vivo Evaluation of the Optimized ISFI Formulation

#### 3.4.1. Pharmacokinetic Parameters

**Figure 12:**
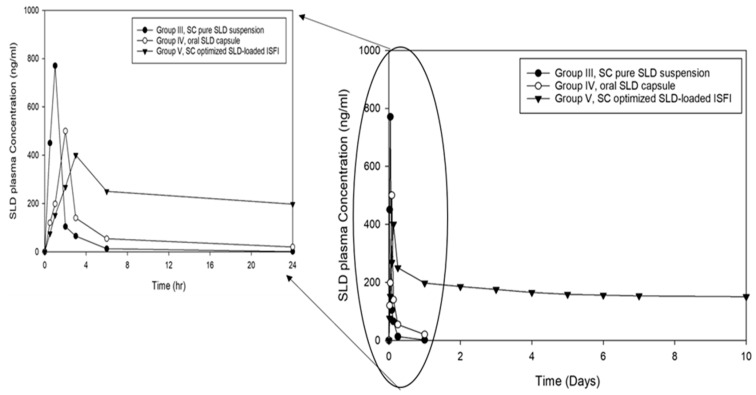
Plasma concentration–time profiles of SC injection of pure SLD, oral administration of commercial SLD capsule, and SC injection of the optimized SLD–ISFI formulation in rats (n = 6) for 10 days (on the right) with the first 24 h magnification (on the left).

The plasma concentration–time profile of SLD was determined after SC injection of pure SLD, oral administration of commercial SLD capsule, and SC injection of the optimized SLD–ISFI formulation, as shown in Figure 12. The pharmacokinetic parameters were calculated and presented in Table 6. The mean C_max_ of the pure SLD was 770.66 ± 5.32 ng/mL, with a mean Tmax of 1.00 ± 0.00 h. The commercial SLD capsule resulted in a mean C_max_ of 500.12 ± 10.05 ng/mL after 2.00 ± 0.01 h. After the SC administration of the optimized SLD–ISFI formulation, the mean C_max_ was 400.37 ± 8.32 ng/mL after 3.00 ± 0.04 h, which was about 1.92-fold lower than the pure SLD and about 1.25-fold lower than the commercial SLD capsule. The optimized SLD–ISFI formulation showed a considerable reduction in the mean C_max_ value compared to the SC pure SLD (*p* < 0.001) and the oral SLD capsule (*p* = 0.004). Additionally, there was a notable increase in the mean t1/2 to 26.14 ± 1.52 h for the optimized SLD–ISFI formulation, compared to 3.84 ± 0.01 h and 8.75 ± 0.11 h for the pure SLD and the commercial SLD capsule, respectively. This suggests the controlled-release impact of the ISFI systems. Also, the AUC_0–∞_ was 13,073.90 ± 67.02 ng/mL.h for the optimized SLD–ISFI formulation, compared to 1190.08 ± 80.24 ng/mL.h and 1996.73 ± 52.12 ng/mL.h for the pure SLD and the commercial SLD capsule, respectively. As shown in Table 6, the optimized SLD–ISFI formulation increased the MRT of SLD by 18.11- and 4.16-fold compared to the pure SLD and the commercial SLD capsule, respectively. The relative bioavailability of SLD was increased by 3.23 when given as SC ISFI compared to the oral commercial SLD capsule (Table 6). This was due to the slowed release of the drug, which led to a delayed removal from the circulation, resulting in improved bioavailability [52].

#### 3.4.2. PSA Measurement

**Figure 13:**
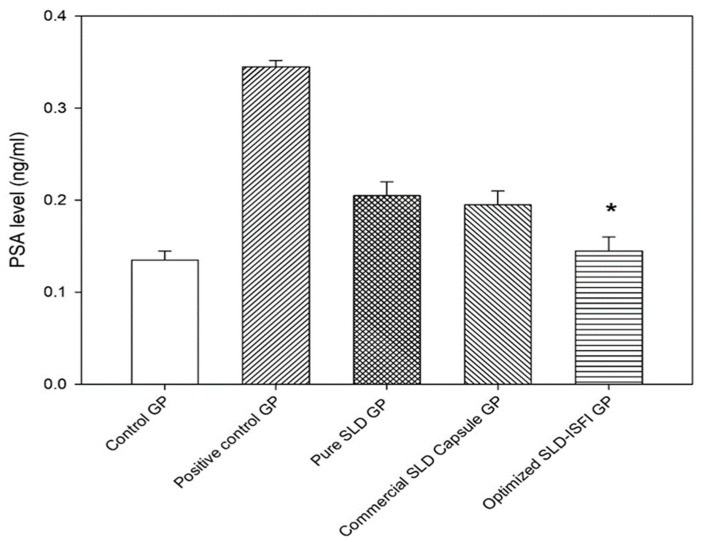
Serum PSA levels in rats after administration of pure SLD, commercial SLD capsule, and optimized SLD-loaded ISFI formulation compared to control and positive control groups. * (*p* < 0.05) in comparison to the positive control group.

PSA is a glycoprotein- and chymotrypsin-like serine protease typically generated in prostate gland cells. Elevated levels of PSA are commonly found in the cancerous cells of men with prostate cancer. The PSA level may also rise in cases of prostatitis and BPH. Therefore, measuring the level of PSA in the serum is utilized as a laboratory test for detecting prostatic hyperplasia in humans [42]. As demonstrated in Figure 13, serum PSA level decreased significantly (*p* = 0.03) from 0.345 ± 0.007 ng/mL in the positive control group to 0.145 ± 0.015 ng/mL with SLD–ISFI formulation. In contrast, pure SLD and the commercial SLD capsule groups showed a non-significant reduction in the serum PSA level to 0.205 ± 0.015 and 0.195 ± 0.015 ng/mL, respectively. The findings suggest that using SLD–ISFI positively impacted the hyperplasic process compared to taking the pure drug and the commercial capsule.

#### 3.4.3. Prostatic Index

After being subjected to the testosterone protocol, the prostatic index in the positive control group (group II) significantly increased by 2.46-fold compared to the control group (group I) (*p* < 0.001) (Table 7). This suggests the protocol successfully induced an experimental BPH model with significant hyperplasia. However, the treatment of rats with pure SLD (group III) (*p* = 0.007) and commercial SLD capsules (group VI) (*p* < 0.001) significantly reduced testosterone-induced increases in the prostatic index by 1.25-fold and 1.68-fold, respectively (Table 7). The group that received the optimized SLD–ISFI formulation (group V) exhibited a significant 2.09-fold reduction in the testosterone-induced increase in the prostatic index (*p* < 0.001). As per Sasidharan et al., an elevated prostatic index is utilized as a significant indicator of BPH development [73]. Hence, the SLD–ISFI formulation’s capacity to decrease the prostatic index of BPH-induced rats convincingly demonstrated the ameliorative potential of the SLD–ISFI compared to the pure drug and the commercial capsule.

#### 3.4.4. Histopathological Evaluation

**Figure 14:**
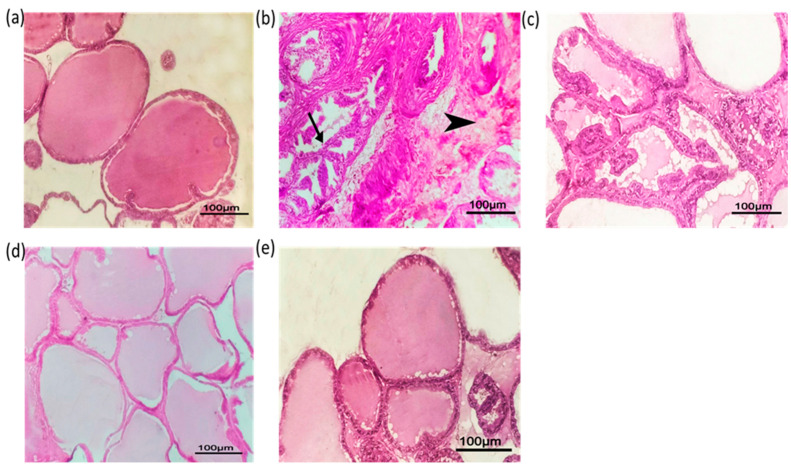
Photomicrographs of H and E sections of male rat ventral lobe of prostate showing (**a**) group I, normal intact regular acini and acinar cells without villous hyperplastic projection; (**b**) group II, the testosterone-treated group with irregular acini with a more high projection inside the lumen which fused to each other (arrow) with focal interstitial fibrosis (arrowhead); (**c**) group III, pure drug-treated group with irregular acini and numerous more wide base projection inside lumen; (**d**) group IV, commercial capsule-treated group with apparently normalized acini without projections and with different sizes; and (**e**) group V, ISFI-treated group with normal intact acini without projections. (Scale bar = 100 µm).

Upon examination, the prostatic tissue sections of the control rats (group I) were morphologically normal. The tissue exhibited an ample quantity of distinctively sized and shaped acini structures (Figure 14a). To evaluate the effects of the different treatments, a reference was established by quantifying the mean number of projections per acini (0.85 ± 0.35) and the mean height of each projection (3.22 ± 0.44 μm) in the control group (Table 7). In contrast, for the positive control group (group II), there was an abnormality found in the tissue structure, which was demonstrated by an increase in cell proliferation, irregular folding and fusion of the acini structures, as well as focal interstitial fibrosis (Figure 14b), with a significant increase in the number of projections (12.00 ± 2.16) (*p* = 0.008) and their height (39.78 ± 9.65 μm) (*p* = 0.013) (Table 7) compared to control. The administration of pure SLD (group III) did not effectively improve the hyperplasia that was present in the testosterone group, with an insignificant reduction in the number of projections to 8.75 ± 0.96 (*p* = 0.083) and height to 30.84 ± 6.32 μm (*p* = 0.099) compared to positive control (Figure 14c, Table 7). However, in Figure 14d, the commercial SLD capsule (group IV) showed apparently normalized acini with significantly fewer intraluminal projections (2.75 ± 1.71) (*p* = 0.020) and lower height (9.14 ± 1.80 μm) (*p* = 0.020) (Table 7) compared to the positive control. In the optimized SLD–ISFI group (Group V), the histoarchitecture of the prostate tissue has also shown signs of improvement. The acini within the tissue appear intact and normal, without any intraluminal projections or fibrous stroma (Figure 14e). The number of acini projections showed a significant reduction to 1.00 ± 0.32 (*p* = 0.008) and a reduction in their height to 3.67 ± 0.59 μm (*p* = 0.013) (Table 7) which were very close to normal. The improvement in prostatic hyperplasia and decreased intraluminal projections compared with the positive control group suggested that the SLD–ISFI formulation displayed an anti-hyperplasic activity and that it is a promising SLD dosage form alternative to oral SLD for the treatment of BPH.

## 4. Conclusions

This study successfully integrated SLD into an injectable ISFI system, resulting in sustained release over 10 days. The use of PLGA polymer in developing SLD-loaded ISFI systems with different organic solvents was explored. Following the I-optimal design, it was demonstrated that increasing the amount of PLGA reduced the initial burst and cumulative release of SLD while increasing the injectability time. A pharmacokinetic study in rats confirmed that the SC administration of SLD–ISFI formulation resulted in lower C_max_ and higher AUC values compared to pure SLD and commercial SLD capsule, indicating the controlled release impact and improved bioavailability of the ISFI systems. Moreover, the optimized SLD–ISFI formulation demonstrated a significant reduction in the testosterone-induced increase in the prostatic index. Finally, histopathological examination of prostatic tissues indicated that the optimized SLD–ISFI formulation exhibited anti-hyperplasic activity, suggesting ISFIs as a promising SLD dosage form alternative to commercial oral SLD for treating BPH.

## Figures and Tables

**Table 1:** Selected independent factors and dependent responses for SLD-loaded ISFI formulations.

Independent Factors	Unit	Symbol	Type	Levels			
**PLGA amount**	mg	A	Numeric	50 (Low)	125 (Medium)		200 (High)
**Type of solvent**	-	B	Categoric	NMP	DMSO	TA	NMP–TA mixture (1:1)
**Dependent responses**	**Unit**	**Symbol**	**Goal**				
**Q_2h_**	%	Y_1_	Minimize				
**Q_10d_**	%	Y_2_	Maximize				
**Injectability time**	sec	Y_3_	Minimize				

**Table 2:** PLGA and SLD solubility in different solvents and their corresponding solubility parameters and molecular volumes.

Solvent	δ (MPa^1/2^) *	Molecular Volume (mL/mol)	SLD Solubility (mg/mL)	PLGA Solubility	Viscosity (mPa s) at 25 °C **
**NMP**	23.1	96.50	674.63 ± 1.12	+++	1.65
**DMSO**	24.5	71.03	580.14 ± 1.39	+++	2.00
**EA**	18.6	97.68	73.40 ± 2.86	+++	0.42
**TA**	23.7	188.2	79.21 ± 1.09	+++	17.4

* obtained from [49]. ** obtained from [50]. +++ means highly soluble.

**Table 3:** Composition of SLD-loaded PLGA ISFIs and observed values of responses.

Run	A	B	Y_1_	Y_2_	Y_3_
	PLGA Amount (mg)	Solvent Type	Q_2h_ (%)	Q_10d_ (%)	Injectability Time (s)
**F1**	125	NMP–TA	44.82	52.94	41.35
**F2**	125	TA	13.69	43.43	110.62
**F3**	125	NMP	66.11	84.26	9.07
**F4**	50	NMP	76.15	88.56	3.42
**F5**	50	NMP–TA	65.17	70.81	10.27
**F6**	50	TA	36.55	53.84	18.95
**F7**	125	DMSO	44.82	62.69	13.14
**F8**	200	NMP–TA	20.32	47.69	76.44
**F9**	125	DMSO	50.79	57.20	18.07
**F10**	50	NMP	66.15	82.49	3.92
**F11**	125	NMP	63.11	80.28	9.94
**F12**	200	NMP–TA	20.32	46.85	71.85
**F13**	200	NMP	57.46	71.61	17.16
**F14**	50	DMSO	56.87	73.99	4.93
**F15**	125	TA	20.36	42.22	104.91
**F16**	200	TA	8.46	26.62	210.75
**F17**	200	DMSO	19.86	44.04	36.54
**F18**	50	DMSO	66.87	69.10	7.37
**F19**	125	NMP–TA	51.85	57.55	35.51

**Table 4:** ANOVA statistical analysis of studied responses.

Terms	Y_1_ (2FI Model)		Y_2_ (Linear Model)		Y_3_ (Quadratic Model)	
	*p*-value	Significance	*p*-value	Significance	*p*-value	Significance
**Model**	<0.0001	Significant	<0.0001	Significant	<0.0001	Significant
**A**	<0.0001	Significant	<0.0001	Significant	<0.0001	Significant
**B**	<0.0001	Significant	<0.0001	Significant	<0.0001	Significant
**AB**	0.0148	Significant	-		<0.0001	Significant
**A^2^**	-		-		0.0106	Significant
**Lack-of-Fit**	0.3728	Not Significant	0.1827	Not Significant	0.4774	Not Significant
**Fit statistics**						
**S.D.**	5.14		3.76		2.88	
**R^2^**	0.9650		0.9621		0.9984	
**Adjusted R^2^**	0.9428		0.9513		0.9970	
**Predicted R^2^**	0.8637		0.9289		0.9926	

ANOVA, analysis of variance; A, PLGA amount; B, type of solvent; Y_1_, burst release after 2 h; Y_2_, cumulative release after 10 days; Y_3_, injectability time; S.D., standard deviation; R^2^, determination coefficient.

**Table 5:** Results of fitting SLD release data from the optimized ISFI formulation to different release kinetic models.

Formulation	Zero Order	First Order	Higuchi	Hixson–Crowell	Korsmeyer–Peppas	
	R^2^	R^2^	R^2^	R^2^	R^2^	n
**Optimized formulation**	0.2395	0.6717	0.9113	0.5699	0.9700	0.233

R^2^, determination coefficient; n, release exponent.

**Table 6:** Pharmacokinetic parameters after administration of SC injection of pure SLD, oral administration of commercial SLD capsule, and SC injection of the optimized SLD–ISFI formulation.

Parameter	SC Pure SLD Suspension	Oral Commercial SLD Capsule	SC Optimized SLD–ISFI
**C_max_ (ng/mL)**	770.66 ± 5.32	500.12 ± 10.05 *	400.37 ± 8.32 *
**T_max_ (h)**	1.00 ± 0.00	2.00 ± 0.01 *	3.00 ± 0.04 *
**K_el_ (h^−1^)**	0.18 ± 0.01	0.08 ± 0.00 *	0.03 ± 0.00 *
**t_1/2_ (h)**	3.84 ± 0.01	8.75 ± 0.11 *	26.14 ± 1.52 *
**AUC_0_** **_–_** **_t_** **(ng/mL·h)**	1182.61 ± 87.87	1741.59 ± 62.04 *	5627.09 ± 54.32 *
**AUC_0_** **_–_** **_∞_** **(ng/mL·h)**	1190.08 ± 80.24	1996.73 ± 52.12 *	13,073.90 ± 67.02 *
**AUMC_0_** **_–_** **_∞_** **(ng/mL·h^2^)**	2613.80 ± 99.02	19,150.83 ± 231.01 *	521,063.99 ± 564.21 *
**MRT (h)**	2.20 ± 0.09	9.59 ± 0.74 *	39.86 ± 2.12 *
**Relative bioavailability (%)**	----	147.27	475.82

* Significant at *p* < 0.05 level in comparison to pure drug suspension. Results are the mean ± standard deviation (n = 6).

**Table 7:** Results of the prostate index (prostate weight/body weight) and histomorphometric parameters after administration of pure SLD, commercial SLD capsule, and optimized SLD-loaded ISFI formulation compared to control and positive control groups (mean ± SD, n = 6).

Groups	Prostatic Index	Histomorphometric Evaluation of Male Rat Ventral Prostatic Lobe	
		No. of Projections/Acini	Projection Height/Acini (µm)
**Group I**	0.28 ± 0.01	0.85 ± 0.35	3.22 ± 0.44
**Group II**	0.69 ± 0.05	12.00 ± 2.16	39.78 ± 9.65
**Group III**	0.55 ± 0.04 *	8.75 ± 0.96	30.84 ± 6.32
**Group IV**	0.41 ± 0.03 *	2.75 ± 1.71 *	9.14 ± 1.80 *
**Group V**	0.33 ± 0.05 *	1.00 ± 0.32 *	3.67 ± 0.59 *

* Significant at *p* < 0.05 level compared to the positive control group.

## Data Availability

The original contributions presented in the study are included in the article further inquiries can be directed to the corresponding author.

## References

[1] Alhayali A., Vuddanda P.R., Velaga S. (2019). Silodosin oral films: Development, physico-mechanical properties and in vitro dissolution studies in simulated saliva. J. Drug Deliv. Sci. Technol..

[2] Kapoor A. (2012). Management in the Primary Care Setting. Can. J. Urol..

[3] Saygisever-faikoglu K., Faikoglu G., Ozcan F.O., Berk B. (2021). The efficacy and safety of silodosin-a review of literature. Pharm. Pharmacol. Int. J..

[4] Sciacqua L.V., Vanzulli A., Di Meo R., Pellegrino G., Lavorato R., Vitale G., Carrafiello G. (2023). Minimally Invasive Treatment in Benign Prostatic Hyperplasia (BPH). Technol. Cancer Res. Treat..

[5] Bakhaidar R.B., Hosny K.M., Mahier I.M., Rizq W.Y., Safhi A.Y., Bukhary D.M., Sultan M.H., Bukhary H.A., Madkhali O.A., Sabei F.Y. (2022). Development and optimization of a tamsulosin nanostructured lipid carrier loaded with saw palmetto oil and pumpkin seed oil for treatment of benign prostatic hyperplasia. Drug Deliv..

[6] Rossi M., Roumeguère T. (2010). Silodosin in the treatment of benign prostatic hyperplasia. Drug Des. Devel. Ther..

[7] Bortnick E., Brown C., Simma-chiang V., Kaplan S.A. (2020). Modern best practice in the management of benign prostatic hyperplasia in the elderly. Ther. Adv. Urol..

[8] Montorsi F. (2010). Profile of Silodosin. Eur. Urol. Suppl..

[9] Montorsi F. (2011). Corrigendum to “Profile of Silodosin” [Eur Urol Suppl 2010;9:491–5]. Eur. Urol..

[10] Jindan L., Xiao W., Liping X. (2022). Evolving Role of Silodosin for the Treatment of Urological Disorders—A Narrative Review. Drug Des. Devel. Ther..

[11] González-Álvarez I., Sánchez-Dengra B., Rodriguez-Galvez R., Ruiz-Picazo A., González-Álvarez M., García-Arieta A., Bermejo M. (2022). Exploring a Bioequivalence Failure for Silodosin Products Due to Disintegrant Excipients. Pharmaceutics.

[12] Ibrahim T.M., El-Megrab N.A., El-Nahas H.M. (2020). Optimization of injectable PLGA in-situ forming implants of anti-psychotic risperidone via Box-Behnken Design. J. Drug Deliv. Sci. Technol..

[13] Chavda V.P., Jogi G., Paiva-santos A.C., Kaushik A. (2022). Biodegradable and removable implants for controlled drug delivery and release application. Expert Opin. Drug Deliv..

[14] Sequeira J.A.D., Santos A.C., Serra J., Veiga F., Ribeiro A.J. (2018). Poly(lactic-co-glycolic acid) (PLGA) matrix implants. Nanostructures for the Engineering of Cells, Tissues and Organs: From Design to Applications.

[15] Major I., Lastakchi S., Dalton M., Mcconville C. (2020). 5. Implantable Drug Delivery Systems.

[16] Kempe S., Mäder K. (2012). In situ forming implants—An attractive formulation principle for parenteral depot formulations. J. Control. Release.

[17] Kerimoglu O., Alarcin E. (2012). Poly(Lactic-Co-Glycolic Acid) Based Drug Delivery Devices for Tissue Engineering and Regenerative Medicine. ANKEM Derg..

[18] Bode C., Kranz H., Fivez A., Siepmann F., Siepmann J. (2019). Often neglected: PLGA/PLA swelling orchestrates drug release: HME implants. J. Control. Release.

[19] Bakhrushina E.O., Sakharova P.S., Konogorova P.D., Pyzhov V.S., Kosenkova S.I., Bardakov A.I., Zubareva I.M., Krasnyuk I.I., Krasnyuk I.I. (2024). Burst Release from In Situ Forming PLGA-Based Implants: 12 Effectors and Ways of Correction. Pharmaceutics.

[20] Zhang X., Yang L., Zhang C., Liu D., Meng S., Zhang W., Meng S. (2019). Effect of polymer permeability and solvent removal rate on in situ forming implants: Drug burst release and microstructure. Pharmaceutics.

[21] Assaf S.M., Ghanem A.M., Alhaj S.A., Khalil E.A., Sallam A.S.A. (2022). Formulation and Evaluation of Eudragit^®^ RL Polymeric Double Layer Films for Prolonged-Release Transdermal Delivery of Tamsulosin Hydrochloride. AAPS PharmSciTech.

[22] Li Z., Mu H., Weng Larsen S., Jensen H., Østergaard J. (2021). An in vitro gel-based system for characterizing and predicting the long-term performance of PLGA in situ forming implants. Int. J. Pharm..

[23] Summonte S., Racaniello G.F., Lopedota A., Denora N., Bernkop-Schnürch A. (2021). Thiolated polymeric hydrogels for biomedical application: Cross-linking mechanisms. J. Control. Release.

[24] Berger J., Reist M., Mayer J.M., Felt O., Peppas N.A., Gurny R. (2004). Structure and interactions in covalently and ionically crosslinked chitosan hydrogels for biomedical applications. Eur. J. Pharm. Biopharm..

[25] Burkoth A.K., Anseth K.S. (2000). A review of photocrosslinked polyanhydrides: In situ forming degradable networks. Biomaterials.

[26] Singh V.K., Pal K., Banerjee I., Pramanik K., Anis A., Al-Zahrani S.M. (2015). Novel organogel based lyotropic liquid crystal physical gels for controlled delivery applications. Eur. Polym. J..

[27] Ahmed T.A., Hussain Z. (2010). Preparation of parenteral in situ gel formulations based on smart PLGA polymers: Concepts to decrease initial drug burst and extend the drug release. Biodegradable Polymers: Recent Developments and New Perspectives.

[28] Song Y., Nagai N., Saijo S., Kaji H., Nishizawa M., Abe T. (2018). In situ formation of injectable chitosan-gelatin hydrogels through double crosslinking for sustained intraocular drug delivery. Mater. Sci. Eng. C.

[29] Sartor O. (2003). Eligard: Leuprolide acetate in a novel sustained-release delivery system. Urology.

[30] Dunn R.L., English J.P., Cowsar D.R., Vanderbilt D.P. (1999). Biodegradable In Situ Forming Implants and Methods of Producing the Same. U.S. Patent.

[31] Ibrahim T.M., Ayoub M.M., El-Bassossy H.M., El-Nahas H.M., Gomaa E. (2022). Investigation of Alogliptin-Loaded In Situ Gel Implants by 23 Factorial Design with Glycemic Assessment in Rats. Pharmaceutics.

[32] Thakur R.R.S., McMillan H.L., Jones D.S. (2014). Solvent induced phase inversion-based in situ forming controlled release drug delivery implants. J. Control. Release.

[33] Pandya A.K., Vora L.K., Umeyor C., Surve D., Patel A., Biswas S., Patel K., Patravale V.B. (2023). Polymeric in situ forming depots for long-acting drug delivery systems. Adv. Drug Deliv. Rev..

[34] Zhao J., Tian G., Qu H. (2023). Application of I-Optimal Design for Modeling and Optimizing the Operational Parameters of Ibuprofen Granules in Continuous Twin-Screw Wet Granulation. Biomedicines.

[35] Ranade S.S., Thiagarajan P. (2017). Selection of a design for response surface. IOP Conf. Ser. Mater. Sci. Eng..

[36] Hegazy D., Tag R., Habib B.A. (2022). Statistical Sequential Experimentation: Preliminary Mixed Factorial Design, I-Optimal Mixture Design Then Finally Novel Design Space Expansion for Optimization of Tazarotene Cubosomes. Int. J. Nanomed..

[37] Tekdemir O., Tilki G., Uğurlu T. (2020). Development of a multiple-unit system: Tablets containing amlodipine besylate which have different release kinetics. J. Res. Pharm..

[38] Öztürk A.A., Banderas L.M., Otero M.D.C., Yenilmez E., Şenel B., Yazan Y. (2019). Dexketoprofen trometamol-loaded poly-lactic-co-glycolic acid (PLGA) nanoparticles: Preparation, in vitro characterization and cyctotoxity. Trop. J. Pharm. Res..

[39] de Abreu L.C.L., de Souza Furtado P., da Silva Honorio T., Hossy B.H., de Pádula M., Domingos T.F.S., do Carmo F.A., de Oliveira Miguel N.C., Rodrigues C.R., de Sousa V.P. (2018). A synergistic nanoformulation of babassu and copaiba oils as natural alternative for prevention of benign prostatic hyperplasia. J. Drug Deliv. Sci. Technol..

[40] Nair A., Jacob S. (2016). A simple practice guide for dose conversion between animals and human. J. Basic Clin. Pharm..

[41] Kishore P. (2012). Validated Estimation of Silodosin in Pure, Pharmaceuticals and in Biological Sample by UV-Spectroscopic and Rp-Hplc Method. Master’s Thesis.

[42] Zhang Y., Huo M., Zhou J., Xie S. (2010). PKSolver: An add-in program for pharmacokinetic and pharmacodynamic data analysis in Microsoft Excel. Comput. Methods Programs Biomed..

[43] Vahabzadeh Z., Molodi M., Nikkho B., Saghebjoo M., Saedmocheshi S., Zamani F., Roshani Y., Babanzadeh S. (2020). Aerobic training and hydroalcoholic extracts of green tea improve pro-oxidant-antioxidant balance and histopathological score in the n-methyl-n-nitrosourea–induced prostate cancer model of rat. EXCLI J..

[44] de Souza Furtado P., Ribeiro da Silva Melo J., Wetler Meireles P., da Silva Honorio T., Campos de Oliveira Miguel N., Simon A., Cunha Sathler P., Coli Louvisse de Abreu L., Almada do Carmo F., Rodrigues C.R. (2021). Benign prostatic hyperplasia therapy through liquisolid technology composed of polymer-layered nanocomposites based on silicate that contain babassu oil and copaiba oil-resin. J. Drug Deliv. Sci. Technol..

[45] Ahmed T., Ibrahim H., Samy A., Kaseem A., Nutan M., Hussain M.D. (2014). Biodegradable injectable in situ implants and microparticles for sustained release of montelukast: In vitro release, pharmacokinetics, and stability. AAPS PharmSciTech.

[46] Camargo J.A., Sapin A., Nouvel C., Daloz D., Leonard M., Bonneaux F., Six J.L., Maincent P. (2013). Injectable PLA-based in situ forming implants for controlled release of Ivermectin a BCS Class II drug: Solvent selection based on physico-chemical characterization. Drug Dev. Ind. Pharm..

[47] Lambert W.J., Peck K.D. (1995). Development of an in situ forming biodegradable poly-lactide-coglycolide system for the controlled release of proteins. J. Control. Release.

[48] Voigt V. (2006). Biodegradable In Situ Forming Systems and Sponge-Like Implants. Ph.D. Thesis.

[49] Barton A.F.M. (1975). Solubility Parameters. Chem. Rev..

[50] Rowe R.C., Sheskey P., Quinn M. (2009). Remington: The Science and Practice of Pharmacy. Handbook of Pharmaceutical Excipients.

[51] Alrashdan M., Shraideh Z.A., Abulateefeh S.R. (2023). Optimizing formulation parameters for the development of carvedilol injectable in situ forming depots. Pharm. Dev. Technol..

[52] Gomaa E., Eissa N.G., Ibrahim T.M., El-Bassossy H.M., El-Nahas H.M., Ayoub M.M. (2023). Development of depot PLGA-based in-situ implant of Linagliptin: Sustained release and glycemic control. Saudi Pharm. J..

[53] Parent M., Nouvel C., Koerber M., Sapin A., Maincent P., Boudier A. (2013). PLGA in situ implants formed by phase inversion: Critical physicochemical parameters to modulate drug release. J. Control. Release.

[54] Calvo N.L., Tejada G., Svetaz L.A., Quiroga A.D., Alvarez V.A., Lamas M.C., Leonardi D. (2021). Development and optimization of a new tioconazole vaginal mucoadhesive film using an experimental design strategy. Physicochemical and biological characterization. J. Pharm. Biomed. Anal..

[55] Ibrahim T.M., Abdulla N.A., Mohamed M.A. (2024). Investigating the efficacy of mirtazapine-embedded invasomal gel nanocarriers via I-optimal design for management of atopic dermatitis. J. Drug Deliv. Sci. Technol..

[56] Bode C., Kranz H., Siepmann F., Siepmann J. (2018). In-situ forming PLGA implants for intraocular dexamethasone delivery. Int. J. Pharm..

[57] Sheshala R., Hong G.C., Yee W.P., Meka V.S., Thakur R.R.S. (2019). In situ forming phase-inversion implants for sustained ocular delivery of triamcinolone acetonide. Drug Deliv. Transl. Res..

[58] Elder S.H., Ross M.K., Nicaise A.J., Miller I.N., Breland A.N., Hood A.R.S. (2024). Development of in situ forming implants for controlled delivery of punicalagin. Int. J. Pharm..

[59] Lin X., Yang S., Gou J., Zhao M., Zhang Y., Qi N., He H., Cai C., Tang X., Guo P. (2012). A novel risperidone-loaded SAIB-PLGA mixture matrix depot with a reduced burst release: Effects of solvents and PLGA on drug release behaviors in vitro/in vivo. J. Mater. Sci. Mater. Med..

[60] Yehia S.A., Elshafeey A.H., Elsayed I. (2012). A novel injectable in situ forming poly-DL-lactide and DL-lactide/glycolide implant containing lipospheres for controlled drug delivery. J. Liposome Res..

[61] Liu H., Venkatranan S.S. (2012). Cosolvent Effects on the Drug Release and Depot Swelling in Injectable In Situ Depot-Forming Systems. J. Pharm. Sci..

[62] Hosny K.M., Rizg W.Y. (2018). Quality by design approach to optimize the formulation variables influencing the characteristics of biodegradable intramuscular in-situ gel loaded with alendronate sodium for osteoporosis. PLoS ONE.

[63] Kanwar N., Sinha V.R. (2019). In situ forming depot as sustained-release drug delivery systems. Crit. Rev. Ther. Drug Carrier Syst..

[64] Abdelnabi D., Lastakchi S., Watts C., Atkins H., Hingtgen S., Valdivia A., McConville C. (2024). Local administration of irinotecan using an implantable drug delivery device stops high-grade glioma tumor recurrence in a glioblastoma tumor model. Drug Deliv. Transl. Res..

[65] Huo L., Wei Y., Zhang H., Wang Y., Deng B., Wang Y., Jin L. (2022). Preparation and properties of triethyl citrate plasticized chitosan-based membranes for efficient release of curcumin. J. Appl. Polym. Sci..

[66] Ibrahim T.M., Eissa R.G., El-Megrab N.A., El-Nahas H.M. (2021). Morphological characterization of optimized risperidone-loaded in-situ gel forming implants with pharmacokinetic and behavioral assessments in rats. J. Drug Deliv. Sci. Technol..

[67] Karimi M., Abrishami M., Farzadnia M., Kamali H., Malaekeh-Nikouei B. (2024). In-situ forming biodegradable implants for sustained Fluocinolone acetonide release to the posterior eye: In-vitro and in-vivo investigations in rabbits. Int. J. Pharm..

[68] Chenna G.P., Sathish Kumar Shetty A., Pai J.B. (2011). Development and validation of RP-HPLC method for quantitative estimation of pyrazinamide in bulk and pharmaceutical dosage forms. Int. J. PharmTech Res..

[69] Lu B., Lv X., Le Y. (2019). Chitosan-modified PLGA nanoparticles for control-released drug delivery. Polymers.

[70] Gaur M., Maurya S., Akhtar M.S., Yadav A.B. (2023). Synthesis and Evaluation of BSA-Loaded PLGA-Chitosan Composite Nanoparticles for the Protein-Based Drug Delivery System. ACS Omega.

[71] He P., Xu S., Guo Z., Yuan P., Liu Y., Chen Y., Zhang T., Que Y., Hu Y. (2022). Pharmacodynamics and pharmacokinetics of PLGA-based doxorubicin-loaded implants for tumor therapy. Drug Deliv..

[72] Mainardes R.M., Gremião M.P.D., Evangelista R.C. (2006). Thermoanalytical study of praziquantel-loaded PLGA nanoparticles. Rev. Bras. Cienc. Farm. J. Pharm. Sci..

[73] Sasidharan S., Srinivasakumar K.P., Bhaumik A., Das S.K., Hareebndran Nair J. (2022). Administration of Caesalpinia bonduc Seed Extracts Ameliorates Testosterone-Induced Benign Prostatic Hyperplasia (BPH) in Male Wistar Rats. Res. Rep. Urol..

